# Comparison Between Size and Stage of Preoperative Tumor Defined by Preoperative Magnetic Resonance Imaging and Postoperative Specimens After Radical Resection of Esophageal Cancer

**DOI:** 10.1177/1533033819876263

**Published:** 2019-09-24

**Authors:** Zhenzhen Gao, Beibei Hua, Xiaolin Ge, Jinyuan Liu, Lei Xue, Fuxi Zhen, Jinhua Luo

**Affiliations:** 1 Department of Clinical Oncology, The Second Affiliated Hospital of Jiaxing University, Jiaxing, China; 2 Department of Radiation Oncology, Yili Friendship Hospital, Xinjiang, China; 3 Department of Radiation Oncology, The First Affiliated Hospital of Nanjing Medical University, Nanjing, China; 4 Department of Thoracic Surgery, The First Affiliated Hospital of Nanjing Medical University, Nanjing, China

**Keywords:** esophageal cancer, magnetic resonance imaging, T staging, pathologic stage, tumor length

## Abstract

**Background:**

Our objective is to explore the accuracy of magnetic resonance imaging in determining the preoperative T and N staging, pathological stage, and the length of esophageal tumor in patients with esophageal cancer.

**Methods:**

This retrospective analysis included 57 patients admitted to the Department of Thoracic Surgery of The First Affiliated Hospital of Nanjing Medical University between January 2015 and December 2016. Postoperative pathological results were used as the reference to verify the accuracy of magnetic resonance imaging in evaluating tumor T and N staging, pathological stage, and tumor length. The correlation between tumor lengths—measured using magnetic resonance imaging and the surgical specimen measurements—was evaluated.

**Results:**

The mean age of the patients was 64.6 ± 7.2 years, with a range of 47 to 77 years. The overall accuracy rate of magnetic resonance imaging in T staging of esophageal cancer was 63.2%; magnetic resonance imaging was generally consistent in the N staging of esophageal cancer. Magnetic resonance imaging and surgical evaluation of tumor length were in excellent agreement (κ = .82, *P* < .001), while that of gastroscopy and postoperative pathology was moderate (κ = .63, *P* < .001).

**Conclusion:**

Magnetic resonance imaging is highly accurate in determining the preoperative T and N staging, pathologic stage, and tumor length in patients with esophageal cancer, which is important in deciding the choice of preoperative treatment and the surgical approach.

## Introduction

The esophagus is clinically divided into the cervical, upper, middle, and lower segments, and the points of distinction are 18, 24, 32, and 40 cm from the incisors, respectively.^1,2^ The esophagus is a mucosal organ that mainly consists of the following 4 layers from inside to outside: mucosa, submucosa, muscles, and outer membrane. As there is no serous membrane around the esophagus, it lies close to the surrounding organs.^3^ Additionally, there is a rich network of lymph nodes around the esophagus, which renders esophageal tumors prone to metastasis. Esophageal cancer—with an insidious onset and high degree of malignancy—is a common malignancy of the digestive tract in China.^4-6^ Surgical resection is the mainstay treatment, and accurate tumor-node-metastasis (TNM) staging is important for selection of the treatment of choice and evaluation of the prognosis. In recent years, there have been several technological advancements in magnetic resonance imaging (MRI) and MRI is widely used in the clinical diagnosis of various diseases. The location of the esophagus in the thoracic cavity is fixed, which limits the degree of dissociation possible. In contrast, artifacts produced during MRI are small. Magnetic resonance imaging equipment has the following types of coils: intracavitary coil, phase-controlled front coil, and extracorporeal coil. Therefore, the presence of esophageal cancer can be examined in multiple locations using MRI.^4,5^ In earlier studies, MRI was not very accurate in diagnosing tumors; however, subsequent studies suggested that combined T2-weighted MRI and diffusion-weighted imaging (DWI) could improve the specificity of MRI in tumor detection. The accuracy of T2-weighted MRI relative to the tumor was 75% to 87% in the original 1.5-T MRI; however, the detection rate of high-resolution MRI in tumor recurrence is unknown.^6^ Furthermore, few studies have reported the use of MRI to examine esophageal cancer in China. Therefore, the aim of this study was to compare the preoperative results of T and N staging using MRI and the postoperative pathology in 57 patients with radical esophageal cancer to explore the clinical diagnostic value of MRI in preoperative staging of esophageal cancer.

## Methods

### General Information

A total of 57 patients who were admitted to the Department of Thoracic Surgery, Jiangsu Provincial People’s Hospital, and underwent radical resection for esophageal cancer between January 2015 and December 2016 were included. All the patients were confirmed to have esophageal squamous cell carcinoma by surgical pathology. Preoperatively, no obvious enlargement of cervical lymph nodes was detected by superficial ultrasound examination and no treatment was administered for the esophageal cancer. All the patients underwent MRI in our hospital preoperatively.

## Methods

### Surgical Procedures

All the surgical procedures were performed with a triple incision of the neck, chest, and abdomen; tubular gastroesophageal substitution; and left neck anastomosis. Thoracic and abdominal lymph nodes were routinely dissected during the surgery.

### Magnetic Resonance Imaging

We used the Philips Achieva 3.0 T magnetic resonance scanner (Germany Siemens Healthcare) with a phase-controlled array coil placed on the body surface. Horizontal, sagittal, and coronal images were obtained using routine turbo spin-echo, T2-weighted scanning (repetition time [TR], 2668 milliseconds; echo time [TE], 80 milliseconds; field of view, 300 × 350; matrix, 544 × 621; layer thickness, 5 mm), axial T1-weighted scanning (TR, 369 milliseconds; TE, 10 milliseconds), and DWI (*b* = 0 and 600 s/mm^2^), and cardiac and respiratory gating were performed. Bayer gadolinium-doped glucosamine injection was used with a 20-mL static push, followed by axial, coronal, and sagittal T1-weighted hyperlipidemic scanning (TR, 508 milliseconds; TE, 10 milliseconds; matrix, 204 × 163). These scanning planes were consistently used in all the patients. When performing axial or coronal scanning, the scanning plane should be parallel or perpendicular to the long axis of the esophagus. In the sagittal-enhanced scan image, the curve measurement technology in Philips 3.0 workstation was used, and the distance between the upper and lower edges of the tumor was defined as the length of the tumor. The average apparent diffusion coefficient (ADC) was calculated from 3 regions of interest, which were artificially chosen in the solid parts of 3 separate layers that contained the tumors. The size and location of an area of interest were selected such that they included as many solid tumor areas as possible. Disagreements were resolved through consensus.

### Diagnostic Criteria

The TNM staging standard for esophageal cancer is based on the eighth edition of the tumor staging system published in 2017 by the American Joint Committee on Cancer/International Union for Cancer Control. Preoperative MRI diagnostic criteria for T staging were as follows: T1, tumor invading the mucosal muscularis or submucosa; T2, tumor invading the muscularis propria; T3, tumor invasion of the outer membrane; and T4, tumor invading adjacent organs, such as the pleura, aorta, or lung. The criteria for metastatic lymph nodes were extrapyramidal or oblong hypoechoic spaces outside the esophagus wall of approximately 1.0 cm diameter with uniform echogenicity and clear or unclear boundaries and medullae. The diagnostic criteria for N staging were as follows: Nx, regional lymph node metastasis cannot be identified; N0, no regional lymph node metastasis; N1, 1 to 2 regional lymph node metastases; N1, 3 to 6 regional lymph node metastases; and N3, more than 7 regional lymph node metastases. Postoperative pathological staging was performed by combining the results of the postoperative pathological examination and the intraoperative findings.

### Statistical Analysis

SPSS version 21 (IBM Corp, Armonk, New York) was used for the statistical analyses. χ^2^ test was used for paired numerical data, and the κ value was used to analyze the agreement of clinical staging between the preoperative MRI and postoperative TNM pathological staging.^7^ Based on κ values of >.75, .75 to .4, and <.4, agreement was categorized as strong, moderate, and weak, respectively. An α level value = .05 and *P* < .05 were considered statistically significant.

## Results

Of the 57 patients, 43 were men and 14 were women. The overall mean age was 64.6 ± 7.2 years, with a range of 47 to 77 years. Overall, 30 and 27 patients had tumors in the middle and lower segments of the esophagus, respectively (Tables 1 and 2). Based on postoperative pathological staging, all patients were of N3 stage and 14, 17, 20, and 6 patients were identified to have stage I, stage II, stage III, and stage IV esophageal cancer, respectively.

**Table 1.** General Information of Enrolled Patients.

Characteristic	Frequency	Percentage
Gender		
Male	43	75
Female	14	25
Age		
<60	13	23
≥60	44	77
Differentiation		
High differentiation	2	3
Intermediate differentiation	33	58
Poor differentiation	22	39
Pathology stage		
I	14	25
II	17	30
III	20	35
IV	6	11
Tumor location		
The upper third	0	0
Middle third	30	53
The lower third	27	47
lymphoma		
0	26	46
1	14	25
2-6	11	19
≥7	6	10

**Table 2.** General Characteristics of Patients and Specific Measurement Data.

Characteristic	Mean ± SD	Range
Age, years	64.6 ± 7.2	47-77
Length (endoscopy), cm	3.67 ± 1.7	1-8
Length (pathology), cm	3.00 ± 1.26	0.3-6.5
Length (MRI), cm	3.64 ± 1.40	1.6-7
ADC value	1.50 ± 0.4	0.71-2.63

Abbreviations: ADC, apparent diffusion coefficient; MRI, magnetic resonance imaging; SD, standard deviation.

**Figure 1.**
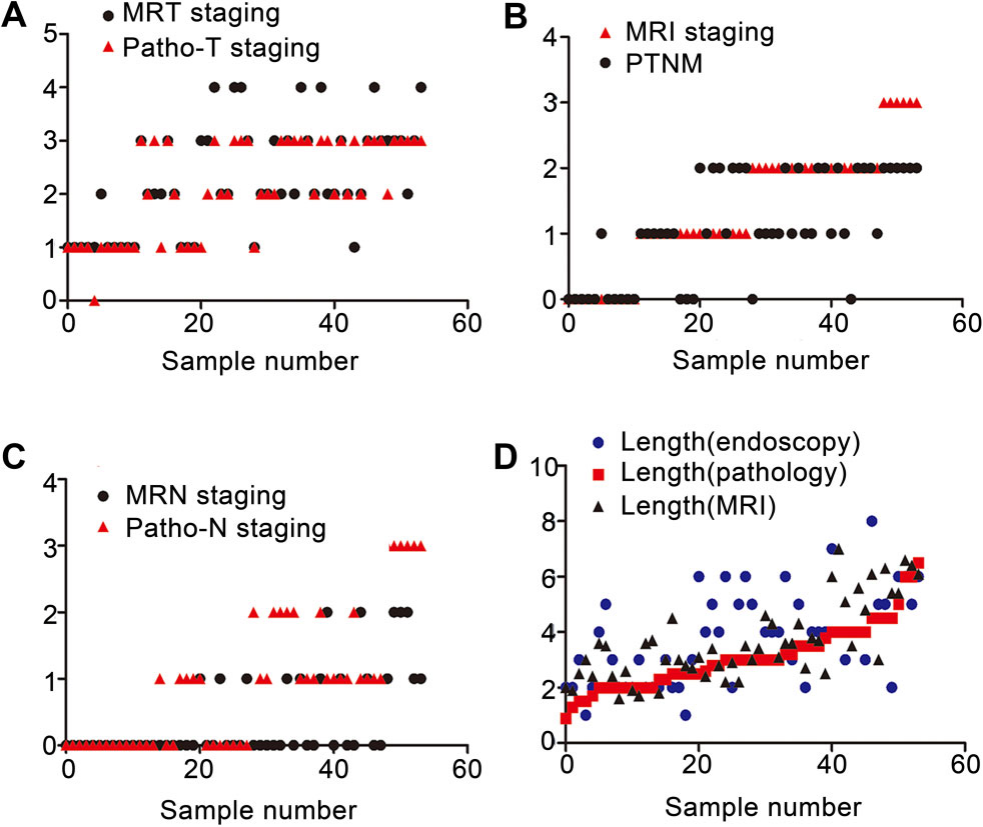
Comparison of MRI and postoperative pathology in the evaluation of T and N staging, pathological stage, and tumor length in patients with esophageal cancer. A, The agreement between MRI evaluation T staging and postoperative T staging was good (κ = .78, *P* < .001). B, MRI stage was generally consistent with postoperative pathological stage (κ =.68, *P* < .001). C, MRI evaluation of N staging was generally consistent with postoperative N staging (κ = .46, *P* < .001). D, The agreement between MRI evaluation of tumor length and postoperative pathological tumor length was good (κ = .71 and *P* < .001), and the agreement between gastroscopy and postoperative pathology was moderate (κ = .63 and *P* < .001). MRI indicates magnetic resonance imaging.

**Figure 2.**
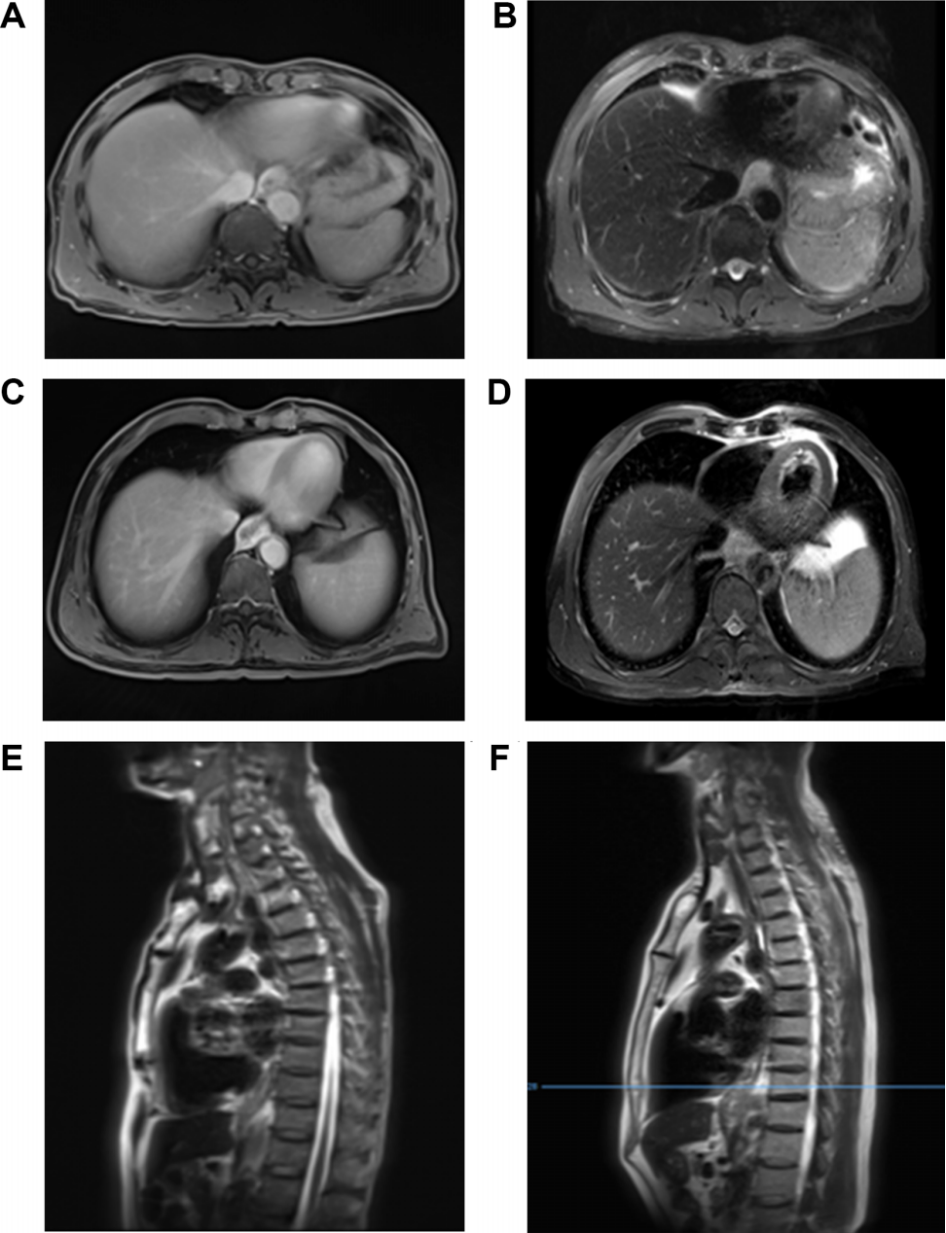
Magnetic resonance images of 2 samples. T2-weighted (A), fat-suppressed T2-weighted (B), MRIs (E) of stage IIB; T2-weighted (C), fat-suppressed T2-weighted (D), MRIs (F) of stage IIIB. MRI indicates magnetic resonance imaging.

The accuracy rate is the ratio of a variable to a population sample. The accuracy of MRI in patients with T1 staging was 83%, including 16 patients with T1 stage and 3 patients with overstaging. The accuracy rate in T2 staging was 77%, with 18 patients with T2 stage and 3 patients with overstaging. The accuracy rate for T3 stage was 42%, with 14 patients with T3 stage, 7 patients with understaging, and 8 patients with overstaging. All the 8 patients with T4 stage were overstaged. Postoperative pathological results revealed 18 patients with Tl, 13 patients with T2, 26 patients with T3, and 0 patients with T4 stages. There were 31 patients with lymph node metastasis and none with distant metastasis. The overall accuracy rate of MRI in T staging of esophageal cancer was 63.2%, which was statistically consistent with the pathological T staging (κ = .78, *P* < .001; Table 3). Magnetic resonance imaging was generally consistent in N staging of esophageal cancer, although there were statistical differences between them (κ = .46, *P* < .001; Table 4). The agreement of tumor length was strong (κ = .82, *P* < .001), while the agreement of combined gastroscopy and postoperative pathology was moderate (κ = .63, *P* < .001). Magnetic resonance imaging showed good agreement in overall TNM staging (κ = .68, *P* < .001; Figure 1). On the basis of aforementioned results, MRIs of 2 patients with stage IIIB (Figure 2A, B, and E) and stage IIB stage (Figure 2C, D, and F) were presented.

**Table 3.** T Staging of MRI and Postoperative Patients.

Pathological Diagnosis	n	MRI Diagnosis				Accuracy Rate (%)
		T1	T2	T3	T4	
T1	18	15	2	1	0	83
T2	13	0	10	3	0	77
T3	26	1	6	11	8	42
Total	57	16	18	14	8	63.2

Abbreviation: MRI, magnetic resonance imaging.

**Table 4.** N Staging of MRI and Postoperative Patients.

Pathological Diagnosis	n	MRI Diagnosis				Accuracy Rate (%)
		N0	N1	N2	N3	
N0	26	25	2	0	0	96
N1	18	12	4	2	0	22
N2	7	5	2	0	0	0
N3	6	0	3	3	0	0
Total	57	42	11	5	0	50.1

Abbreviation: MRI, magnetic resonance imaging.

Furthermore, we evaluated the relationship between ADC and the prognostic factors. In all patients, the average ADC was 0.44 ± 0.096 mm^2^/s. Table 5 summarizes the differences in ADCs before treatment between the groups. There was a significant difference in the average ADCs between the N stage group and the pathological stage group; the higher the T stage, the greater was the lymph node involvement and lower was tumor differentiation ADCs. On MRI, there were statistically significant differences in the T staging, N staging, and pathological grade.

**Table 5.** Relationship Between Preoperative ADC and Preoperative Imaging Stage and Postoperative Pathology.

Factor	Classification	n	ADC Value	*P* Value
MRT staging	T1-2	32	0.15 ± 0.08	.001
	T3-4	25	0.84 ± 0.18	
MRN Staging	N0	38	0	.0001
	N+	19	1.47 ± 0.10	
Tumor grade	Well differentiated	2	0	.001
	Moderately differentiated	32	0.33 ± 0.10	
	Poorly differentiated	23	0.61 ± 0.18	

Abbreviations: ADC, apparent diffusion coefficient; MR, magnetic resonance.

## Discussion

Esophageal cancer, which ranks sixth in terms of overall mortality or mortality in cancer in the world, is a refractory tumor with poor prognosis and includes esophageal squamous cell carcinoma and esophageal adenocarcinoma.^8,9^ In 2012, an epidemiological survey found that esophageal squamous cell carcinoma accounts for 88% of esophageal cancers. Furthermore, there are large regional differences in incidence of esophageal cancer, with higher incidences in eastern Asia, central Asia, and the East African Rift System.^10,11^ The incidence of esophageal cancer in China is concentrated in the Taihang Mountains. Once esophageal cancer is diagnosed, surgery is the treatment of choice.^12-16^ If a patient is diagnosed with locally advanced esophageal cancer, preoperative chemotherapy or radiotherapy is necessary.

Magnetic resonance imaging was used to identify invasions of T1 and T2 stage tumors. If a lesion was confined to the mucosa and submucosa, it implied that the muscle layer was not invaded, which was a stage T1 sign. At present, there is no significant difference in the treatment of T1 and T2 tumors; however, the treatment of T3 tumors is quite different. Therefore, accurate diagnosis and distinction of T2 and T3 esophageal cancer is very important.^17,18^ Based on the analysis of stage T2 and T3 tumors in this study, the main differentiating points between these tumors are that T3 tumors involve the muscularis and intestinal wall with loss of surrounding fat boundaries, can be characterized by a muscle layer that is incomplete or interrupted, esophagus appears to be dispersed in the adipose tissue around the base of soft tissue, and the cavity of the tumor has the same signal characteristics, which is a reliable sign of tumor invasion into the surrounding adipose tissue. The key points to differentiate T3 stage from T4 stage tumors are that T4 stage tumors invade the adjacent organs and that there is no adipose layer between the tumors and peripheral organs. Diffusion-weighted imaging sequences demonstrate high signal of apparent diffusion restriction—similar to that of the tumor tissue—in adjacent organs.^17,19^ The results of this study demonstrate that the accuracy of MRI in identifying T1 and T2 tumors was high but was significantly lower for T3 tumors, suggesting the limitations of MRI in determining stages T3 and T4. It was difficult to clearly distinguish peripheral inflammatory hyperplasia and tumor lesions, and therefore the ability to distinguish between T2 and T3 stages of esophageal cancer was poor. The detection of lymph nodes has a decisive effect on the diagnosis and treatment of esophageal cancer. Additionally, the prognoses in patients with positive lymph nodes were significantly worse than those with negative lymph nodes.^20,21^ In earlier studies, the size of the lymph nodes was used as a positive indicator of lymph node metastasis; however, previous studies have demonstrated that enlarged lymph nodes do not always indicate metastasis and may result from exaggerated inflammatory response, whereas lymph nodes of normal size can also have micrometastases.^22^ Therefore, the size of lymph nodes is not the standard to distinguish benign and malignant lesions. The accuracy in this study for evaluating lymph node metastasis was 50.1%. Therefore, MRI demonstrated poor agreement between the assessment of metastatic lymph nodes and the pathologically confirmed metastatic lymph nodes.

In MRI, DWI sequence is a simple and easy inspection method. Apparent diffusion coefficient is used not only for the detection and characterization of tumors but also for the detection of tumor treatment response.^23^ The ADC value reflects the dispersion characteristics of water molecules, which are determined by multiple factors such as cell density, vascular distribution, viscosity of liquid, and permeability of cell membrane.^24,25^ In our study, the average preoperative ADC value was higher in tumors with lymph node metastases than that in those without lymph node metastases. Therefore, it turns out that lymph node metastasis is a powerful predictor of distant metastasis. Additionally, the correlation between ADC value and lymph node involvement implies that the ADC value is associated with the prognosis. Considering that the ADC value is estimated indirectly based on the microscopic movements of the tumor cell structures, it can reflect the invasiveness of the tumor tissue. The relatively high ADC value of poorly differentiated tumors further confirms this view; therefore, a low ADC value indicates higher malignancy in tumors. A recent study also demonstrated that a higher ADC value represents less differentiated tumors.^26,27^ In our study, we confirmed the correlation between preoperative MRI staging/postoperative pathology and ADC values using χ^2^ test. The higher the T stage, the greater was the ADC value; the worse the differentiation degree, the greater was the ADC value. These findings demonstrate that ADCs can be used as imaging indicators that reflect the biological characteristics in esophageal cancer.

To date, only few studies have applied preoperative MRI staging in the evaluation of esophageal cancer. Giganti *et al* compared the specificity and accuracy of MRI/multidetector computed tomography (CT)/positron emission tomography-CT in T staging. The limitations of each of them were described, and it was further proved that the ADC of MRI-DWI combined with T2-weighted staging could be better used for T staging.^28^ Our study further analysis the utility of MRI in predicting the TNM staging. Magnetic resonance imaging can accurately evaluate the infiltration depth and lymph node metastasis in esophageal cancer; its multidirectional imaging features can be used to evaluate the preoperative T and N stages along with the relationship between the tumor and adjacent organs. Additionally, ADC value was correlated with the degree of tumor differentiation and invasiveness in this study. In conclusion, MRI has good application potential and value in the diagnosis of esophageal cancer, especially the calculation of ADC value combined with T2-weighted staging, which may assist clinicians to make better preoperative judgment.

## Abbreviations

ADC: apparent diffusion coefficient
CT: computed tomography
DWI: diffusion-weighted imaging
MRI: magnetic resonance imaging
TE: echo time
TNM: tumor-node-metastasis
TR: repetition time.
